# Bioengineered Lacrimal Gland Organ Regeneration *in Vivo*

**DOI:** 10.3390/jfb6030634

**Published:** 2015-07-30

**Authors:** Masatoshi Hirayama, Kazuo Tsubota, Takashi Tsuji

**Affiliations:** 1 Department of Ophthalmology, Keio University School of Medicine, Shinjuku-ku, Tokyo 160-8582, Japan; E-Mails: mar_hirayama@z2.keio.jp (M.H.); tsubota@z3.keio.jp (K.T.); 2 Laboratory of Organ Regeneration, RIKEN Center for Developmental Biology, Kobe, Hyogo 650-0047, Japan; 3 Organ Technologies Inc., Chiyoda-ku, Tokyo 101-0048, Japan

**Keywords:** lacrimal glands, regenerative medicine, 3D cell manipulation, organ regeneration, dry eye disease

## Abstract

The lacrimal gland plays an important role in maintaining a homeostatic environment for healthy ocular surfaces via tear secretion. Dry eye disease, which is caused by lacrimal gland dysfunction, is one of the most prevalent eye disorders and causes ocular discomfort, significant visual disturbances, and a reduced quality of life. Current therapies for dry eye disease, including artificial tear eye drops, are transient and palliative. The lacrimal gland, which consists of acini, ducts, and myoepithelial cells, develops from its organ germ via reciprocal epithelial-mesenchymal interactions during embryogenesis. Lacrimal tissue stem cells have been identified for use in regenerative therapeutic approaches aimed at restoring lacrimal gland functions. Fully functional organ replacement, such as for tooth and hair follicles, has also been developed via a novel three-dimensional stem cell manipulation, designated the Organ Germ Method, as a next-generation regenerative medicine. Recently, we successfully developed fully functional bioengineered lacrimal gland replacements after transplanting a bioengineered organ germ using this method. This study represented a significant advance in potential lacrimal gland organ replacement as a novel regenerative therapy for dry eye disease. In this review, we will summarize recent progress in lacrimal regeneration research and the development of bioengineered lacrimal gland organ replacement therapy.

## 1. Introduction

Advances in regenerative medicine, influenced by our understanding of developmental biology, stem cell biology, and tissue engineering, are expected to underlie next-generation medical therapies [1,2,3]. Regenerative medicine for various organs, such as stem cell transplants of enriched or purified tissue-derived stem cells and cytokine therapies that activate tissue stem cell differentiation, have been clinically developed and applied [4,5]. These therapies represent attractive concepts with the potential to partially restore lost organ functionality in damaged tissues, malignant diseases, myocardial infarction, neurological diseases, and hepatic dysfunction [6,7,8,9]. Current tissue engineering technologies have established two-dimensional tissue regeneration approaches, including the cell sheet transplant technique [10]. The concept of regenerative medicine in ophthalmology includes corneal limbal stem cell transplants, which are based on the understanding of stem cell biology, and regenerative cell sheets, such as cultivated corneal epithelial cell sheets and cultivated oral mucosal epithelial cell sheets, and this has contributed to effective ocular surface reconstruction in clinics for severe ocular surface disorders [11,12,13]. Regenerative therapies in ophthalmology have steadily advanced to overcome vision-threatening eye diseases, including those of the cornea and retina [14].

Clinically transplanting donor organs is an important therapeutic approach for severe organ dysfunctions; however, there are related medical issues, including allogenic immunological rejection and critical donor shortage [15]. The use of fully functional substitute organs, including artificial organs made from mechanical devices and bio-artificial organs, which consist of living cells and artificial polymers, has been demonstrated to reproduce physiological functions for various organs [16,17,18,19]. Organ replacement regenerative therapy for tissue repair, via reconstruction of a fully functional, bioengineered organ from stem cells using *in vitro* three-dimensional cell manipulation, is one of the ultimate goals for regenerative medicine: the replacement of dysfunctional organs arising from disease, injury, or aging [20]. Developing cell manipulation techniques *in vitro*, through the precise arrangement of several different cell species and organ culture methods, is required to realize the next generation of three-dimensional, functional, bioengineered organ replacement regenerative therapy [21].

This review details the physiological functions, diseases, and development of the lacrimal gland obtained from published stem cell research. We illustrate that there is potential for novel, fully functional lacrimal gland regeneration as a next-generation regenerative medicine [22,23].

## 2. Physiological Function of the Lacrimal Glands

The lacrimal glands are essential for maintaining the physiological function and homeostasis of the ocular surface microenvironment via tear secretion [24,25]. The lacrimal gland consists of the main lacrimal gland, which primarily secretes aqueous tears, and small accessory lacrimal glands [25]. Mature lacrimal glands are organized into a tubuloalveolar system, which includes the acini, the ducts that carry fluid from the acini to a mucosal surface, and the myoepithelial cells that envelop the acini and early duct elements [25]. For physiological tear secretion, establishing the secretagogue stimulus-secretion coupling mechanisms and innervation is required. A tear film consisting of lipid, aqueous, and mucin layers contributes to the microenvironment homeostasis and optical properties of the ocular surface [26,27,28,29,30]. The aqueous layer of the tear film is secreted by the lacrimal glands and contains water and various tear proteins, such as lactoferrin, with biological functions including moisturizing capacity and antimicrobial activity [31,32,33,34,35,36]. The lacrimal gland and tear functions are indispensable in protecting the epithelial surface and visual function.

## 3. Dry Eye Disease

Dry-eye disease (DED) is caused by a tear shortage due to lacrimal gland dysfunction that results from systemic diseases and environmental exposures, such as Sjogren’s syndrome and ocular cicatricial pemphigoid, or other causes, including aging, long-term work with visual displays, the use of contact lenses, low-humidity environments, and refractive surgery [37,38,39,40,41,42,43,44,45,46,47,48,49]. DED is one of the most common eye diseases, and it causes ocular surface epithelial damage, which leads to ocular discomfort, significant loss of vision, and a reduced quality of life [12,50,51]. Current therapies for DED, such as artificial tear solutions, are palliative and do not completely substitute normal tear complexes that contain water, salts, hydrocarbons, proteins, and lipids [52,53,54]. A therapeutic approach using regenerative medicine is expected to restore lacrimal gland function as a cure for DED [55].

## 4. Organogenesis of the Lacrimal Glands

**Figure 1:**
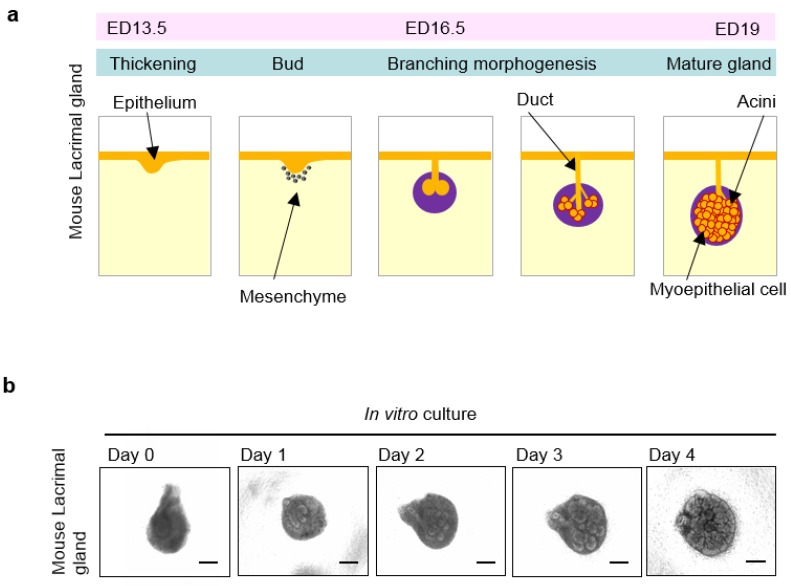
Lacrimal gland organogenesis via epithelial-mesenchymal interactions: (**a**) Schematic representation of the lacrimal gland development during embryogenesis; (**b**) Phase-contrast images of the *in vitro* lacrimal gland germ organ culture development. Scale bars, 100 µm. Modified and reprinted from Hirayama *et al.* [23].

Organs, including the lacrimal glands, are functional units composed of various cells with the appropriate three-dimensiona histological architecture, which is achieved through developmental processes in the embryo, to work efficiently. Almost all ectodermal organs, such as teeth, hair follicles, and lacrimal glands, exhibit similar embryonic development from their organ germs that involves reciprocal epithelial and mesenchymal interactions [56]. Branching morphogenesis, which is a fundamental process for developing lacrimal glands, leads to the specification of the ocular surface epithelium and the induction of the lacrimal gland germ (Figure 1a,b) [57,58]. The development of the murine lacrimal gland occurs on embryonic day (ED) 13.5 via a tubular invagination of the conjunctival epithelium at the temporal region of the eye [59]. After the epithelium invaginates and elongates, the lacrimal gland germ invades the mesenchymal sac on ED 16.5 and begins to rapidly proliferate and branch to form a lobular structure [59,60,61,62]. The development of lacrimal gland structures is essentially completed by ED 19. By the time the eyes open, seven days after birth, secretory tear components including proteins and lipids are produced [63,64]. Mouse harderian glands, which secrete lipids, also play an important role in protecting the ocular surface [65]. The harderian glands originate from the nasal region of the conjunctival epithelium at ED 16 via a developmental branching process similar to that of the lacrimal glands, and they are located behind the eye [65,66]. The harderian glands are either degenerated or do not exist in primates, including humans [65]. This comprehensive developmental mechanism, involving branching morphogenesis, modulates lacrimal gland maturation.

## 5. Tissue Stem Cells in the Lacrimal Gland

To restore lacrimal gland function, several therapeutic approaches have been reported, such as ectopic salivary gland transplantation *in vivo* [67,68] and regenerative medicine [69]. Secretory glands, including salivary glands, the pancreas [70,71], and mammary glands [72], can self-renew after tissue injury, and this process is mediated by tissue stem cells. Many studies aimed at restoring secretory gland function have attempted to use various stem cells derived from adult tissues [73,74]. For salivary glands, long-term abnormal ligation of the salivary excretory duct leads to inflammation and cell death, which results in gland atrophy; however, some repair processes, including the proliferation of the tubuloalveolar structure, do occur when the ligation is released [75,76,77,78,79,80,81]. The salivary gland can potentially regenerate using various stem cells, such as intercalated duct cells from the salivary gland [76], c-kit-positive duct cells in human salivary glands [75], salivary gland-derived progenitor cells isolated from duct-ligated animals, and bone marrow-derived Sca-1- and c-kit-positive cells [73]. For stem cell therapy of the lacrimal glands, the potential existence of stem cells or progenitor cells has been previously described [69,82]. Tissue stem/progenitor cells, which express nestin and Ki67, and mesenchymal cells both contribute to tissue repair after interleukin-1-induced inflammation in murine-lacrimal glands [83,84,85,86]. Stem cell candidates expressing stem cell markers such as c-kit, ABCG2, and ALDH1 have been identified in human lacrimal gland cells [87,88]. Tissue regeneration using transplanted stem cells in adult tissues to restore lacrimal gland function is an area of intense research because of its potential clinical benefits [89,90].

## 6. A Novel Three-Dimensional Cell Manipulation Method Termed the Organ Germ Method

**Figure 2:**
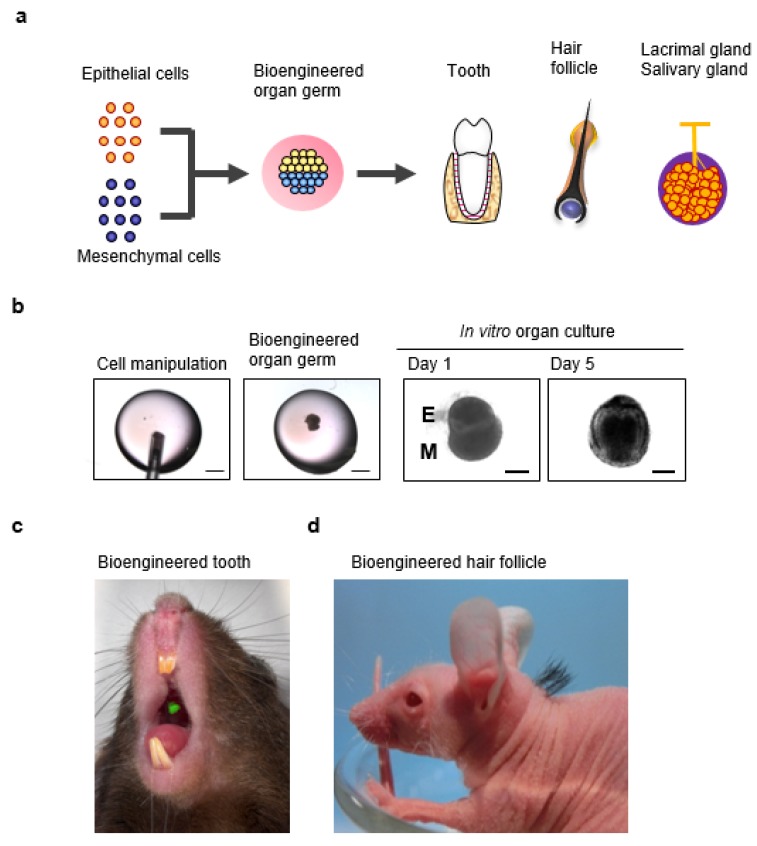
Strategy for bioengineered organ regeneration using the organ germ method: (**a**) Functional organs, such as teeth, hair follicles, salivary glands, and lacrimal glands, can now be regenerated *in vivo* by transplanting bioengineered organ germs reconstituted from epithelial and mesenchymal cells via the organ germ method; (**b**) Representative image of our developed three-dimensional cell processing system, the organ germ method. A high density of dissociated mesenchymal cells is injected into the center of a collagen drop (left panel). Dissociated epithelial cells are subsequently injected into the drop adjacent to the mesenchymal cell aggregate (center-left panel). The bioengineered tooth regenerated via the organ germ method could develop into an appropriate tooth germ via organ culturing (center-right and right panels); (**c**) Photograph showing the green fluorescence protein (GFP)-labeled bioengineered tooth engrafted in an adult mouse (green); (**d**) Photograph of the developed bioengineered hair follicles, which were successfully engrafted into a nude mouse. Modified and reprinted from Nakao *et al.* [21].

To further these biological technologies, the development of methods for the manipulation of multiple cells is required to realize three-dimensional organ regeneration for functional bioengineered organ replacement therapy [20]. A novel strategy for developing bioengineered organs by reproducing the developmental process during organogenesis has been proposed for the functional replacement and complete restoration of lost organs [21]. This bioengineered organ germ method, which manipulates epithelial and mesenchymal cells via cell compartmentalization at a high cell density in a type I collagen gel matrix, was developed to reconstruct bioengineered organ germs *in vitro* as an organ engineering technology (Figure 2a,b) [91,92]. This method successfully developed bioengineered ectodermal organs, such as teeth and hair follicle germs, through multicellular assembly and epithelial and mesenchymal interactions similar to those in natural organ germs (Figure 2c,d) [91,92,93,94,95]. Importantly, the bioengineered tooth and hair follicle germ transplants could restore physiological functions via cooperation with peripheral tissues at the lost tooth or hair follicle [93,94,95,96]. Developing this method was a substantial advance towards potentially regenerating other ectodermal secretory organs, including the salivary glands [97,98] and lacrimal glands [23].

## 7. Fully Functional Lacrimal Gland Organ Regeneration

**Figure 3:**
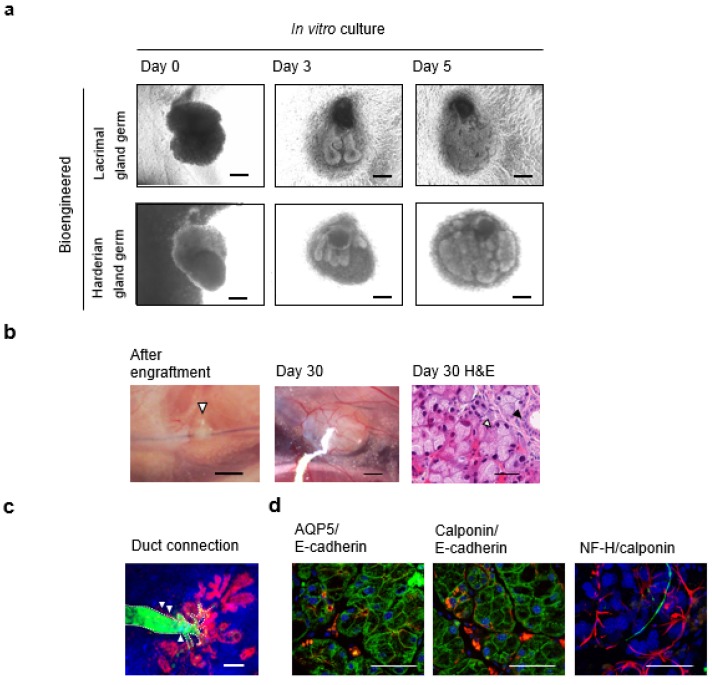
Functional lacrimal gland regeneration via bioengineered lacrimal gland germ transplant: (**a**) Phase-contrast images of the bioengineered lacrimal gland germ (upper line) and bioengineered harderian gland germ (lower line) development. Scale bar, 100 µm; (**b**) Photographs of the bioengineered lacrimal gland germ after transplanting into a mouse with the extra-orbital lacrimal gland removed (arrowhead) (left panel; Scale bar, 1 mm). At 30 days after transplantation, the bioengineered lacrimal gland was successfully engrafted and developed (center; Scale bar, 500 µm). The hematoxylin-eosin(H.E.) staining revealed that the bioengineered lacrimal gland achieved a mature secretory gland structure including acini (white arrowhead) and duct (black arrowhead) (right; Scale bar, 50 µm); (**c**) Histological analysis of the duct connection between the bioengineered lacrimal gland and recipient lacrimal excretory duct. Bioengineered lacrimal glands regenerated using DsRed transgenic mouse-derived epithelial cells (red) and normal mouse-derived mesenchymal cells developed with the correct duct association in the recipient mouse (arrowhead). Fluorescein isothiocyanate (FITC) -gelatin (green), which was injected from the recipient lacrimal excretory duct, could successfully reach the bioengineered lacrimal gland. 4',6-diamidino-2-phenylindole (DAPI; blue) and the excretory duct (dotted line) are shown. Scale bars, 100 µm; (**d**) Immunohistochemical analysis of the bioengineered lacrimal gland after transplantation. Aquaporin-5 is red and E-cadherin is green in the left panel. Calponin is red and E-cadherin is green in the center panel. Calponin is red, neurofilament-H (NF-H) is green, and DAPI is blue in the right panel. Scale bars, 50 µm. Modified and reprinted from Hirayama *et al.* [23].

We investigated whether our organ germ method could regenerate a bioengineered lacrimal gland and restore its physiological function. The bioengineered lacrimal gland germ, which was reconstituted using the epithelial and mesenchymal cells from the lacrimal gland germ of an ED 16.5 mouse, successfully developed branching morphogenesis followed by stalk elongation and cleft formation in organ culture *in vitro*. Bioengineered harderian gland germs were also regenerated via the organ germ method (Figure 3a).

### 7.1. Engraftment of Bioengineered Lacrimal Gland Germ with Duct Association

A duct association between the bioengineered lacrimal gland and the mouse receiving the ocular surface discharge is required for tear secretion from the bioengineered lacrimal gland. The bioengineered lacrimal gland germ and the bioengineered harderian gland germ were successfully engrafted to a mouse from which an extra-orbital lacrimal gland had been removed, and the bioengineered lacrimal gland duct was connected to the recipient lacrimal excretory duct using our thread-guided transplant method (Figure 3b,c). After the transplant, the bioengineered lacrimal and harderian glands formed the appropriate histo-architecture, including acini-expressing aquaporin 5 and myoepithelial cells, duct, and nerve fibers, by reproducing the developmental process that occurs during organogenesis (Figure 3c,d). Thus, the bioengineered lacrimal gland and harderian gland can develop after *in vivo* orthotopic or ectopic transplantation.

### 7.2. Tear Secretion Ability of the Bioengineered Lacrimal Gland

**Figure 4:**
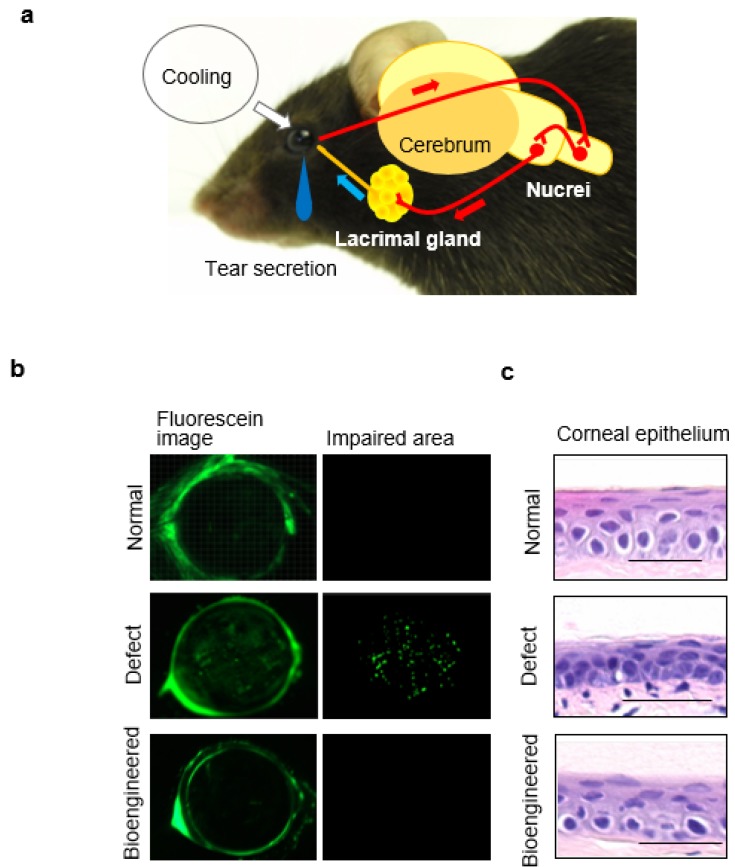
Tear secretion and ocular surface protection for the bioengineered lacrimal gland: (**a**) Schematic representation of the neural reflex loop for tear secretion. Cooling on the ocular surface stimulates tear secretion from the lacrimal gland via the central nervous system; (**b**) Representative images of the corneal surface of a normal lacrimal gland (upper), a lacrimal gland-defect mouse (center), and a bioengineered lacrimal gland–engrafted mouse (lower). The punctate staining area by fluorescein showed impaired area on corneal surface. Scale bar, 1 mm. Modified and reprinted from Hirayama *et al.* [23]; (**c**) Representative microscopic images of the corneal epithelium, including a normal mouse (upper), lacrimal gland–defective mouse (center), and bioengineered lacrimal gland–transplanted mouse (lower) are shown. Chronic dry eye status in lacrimal gland–defective mouse induced corneal thickening as shown in the center panel, whereas these changes were not observed in the bioengineered lacrimal gland-transplanted mouse. Scale bars, 25 μm. Modified and reprinted from Hirayama *et al.* [23].

Reconstituting neural pathways between the bioengineered lacrimal gland and the recipient’s neural system is important to protect the ocular surface via restored tear secretion [99,100,101]. Tearing resulting from a cooling stimulation at the ocular surface that is activated via corneal thermoreceptors and is a representative neural pathway for lacrimal gland function (Figure 4a) [102,103]. We demonstrated that the bioengineered lacrimal gland could secret tears in cooperation with peripheral tissues, including neural systems, because the tear secretion volume from the bioengineered lacrimal gland increased after ocular surface cooling stimulation. Tear components secreted from acini in the lacrimal and harderian glands, such as lactoferrin and lipids, respectively, are essential for physiological tear functions such as increased stability, wound healing, and anti-bacterial effects [104,105,106,107,108,109]. Current therapies for severe lacrimal gland dysfunction include medical treatments such as albumin eye drops and autologous serum eye drops that attempt to substitute tear protein function [54,110,111,112,113,114]. We have shown that tears from the bioengineered lacrimal gland contained major tear proteins, including lactoferrin. In addition, the lipid concentration increased significantly in tears from the bioengineered harderian gland. These results indicated that these bioengineered glands can produce appropriate tear components. Functional replacements of the bioengineered lacrimal gland would be an attractive strategy for treating severe dry eye disease.

### 7.3. Ocular Surface Protection Effect by the Bioengineered Lacrimal Gland

Protecting the ocular surface is the main purpose of using the bioengineered lacrimal gland to restore lacrimal gland function. Punctate staining of the impaired area on the ocular surface [115,116] and corneal epithelial changes including thinning and stromal fibroblast activation [117,118] were observed in a mouse with an extra-orbital lacrimal gland defect, which mimics the corneal epithelial damage caused by lacrimal gland dysfunction. However, these changes were prevented using a bioengineered lacrimal gland (Figure 4b,c). Our results indicate that the bioengineered lacrimal gland can develop and provide sufficient function to maintain a healthy ocular surface.

## 8. Conclusions and Future Directions

Bioengineered lacrimal gland germs exhibit appropriate physiological functions, such as tear secretion, in response to nervous stimulation and ocular surface protection. These studies are a proof-of-concept for bioengineered organs that can functionally restore the lacrimal gland. Our bioengineered organ regeneration concept, which has also been applied to salivary gland regeneration [98], provides substantial advances for regenerative therapies for dry eye disease and xerostomia. Epithelial and mesenchymal stem cells, which have organ-inductive potential for bioengineered organs, have not been reported in adult tissues. To realize the future practical clinical applications of organ replacement regenerative therapy, studies to develop technologies for organ regeneration, such as investigations of available cell sources (e.g., pluripotent stem cells represented as embryonic stem cells and induced pluripotent stem cells) and the efficacy of disease models (e.g., Sjogren syndrome and Stevens-Johnson syndrome) for these methods, technical procedures for culture methods to create bioengineered organs, and appropriate transplantation methods for human patients, are required. Bioengineered organ regenerative therapy is expected to be an essential therapeutic strategy for the next generation of regenerative medicine.
